# Fatty Acid-Binding Protein 7 (FABP-7), Glutamic Acid and Neurofilament Light Chain (NFL) as Potential Markers of Neurodegenerative Disorders in Psoriatic Patients—A Pilot Study

**DOI:** 10.3390/jcm11092430

**Published:** 2022-04-26

**Authors:** Julia Nowowiejska, Anna Baran, Justyna Magdalena Hermanowicz, Beata Sieklucka, Julita Anna Krahel, Paulina Kiluk, Dariusz Pawlak, Iwona Flisiak

**Affiliations:** 1 Department of Dermatology and Venereology, Medical University of Bialystok, Zurawia 14 St., 15-540 Bialystok, Poland; anna.baran@umb.edu.pl (A.B.); julita.leonczuk@gmail.com (J.A.K.); paulina.kiluk@umb.edu.pl (P.K.); iwona.flisiak@umb.edu.pl (I.F.); 2 Department of Pharmacodynamics, Medical University of Bialystok, Mickiewicza 2C St., 15-540 Bialystok, Poland; justyna.hermanowicz@umb.edu.pl (J.M.H.); beata.sieklucka@umb.edu.pl (B.S.); dariusz.pawlak@umb.edu.pl (D.P.)

**Keywords:** fatty acid-binding protein 7, FABP 7, brain fatty acid-binding protein, glutamic acid, glutamate, neurofilament light polypeptide, NFL, psoriasis, neurodegenerative disease, Alzheimer’s disease, Parkinson’s disease

## Abstract

Psoriasis and neurodegenerative diseases (NDs) are important medical, social and economic issues. The possible relationship of psoriasis and NDs has not been established yet. This study involved 60 patients with plaque-type psoriasis. Serum concentrations of fatty acid-binding protein 7 (FABP-7), glutamic acid (GA) and neurofilament light chain (NFL), which have been hardly studied in psoriasis before, were measured by ELISA before and after 12 weeks of treatment with acitretin or methotrexate. The concentration of FABP-7 and NFL in patients before the treatment was significantly higher than in the controls (*p* < 0.01, *p* < 0.001, respectively). After the treatment their concentration decreased, although FABP-7 did so insignificantly. The concentration of GA did not differ significantly between patients and controls and before and after the treatment but we found its negative correlation with CRP (*p* < 0.05). The duration of psoriasis does not seem to directly affect the risk of neurodegeneration and the severity only in patients with worse skin condition. Elevated FABP-7 and NFL, which are present in the brain, may be considered as potential indicators of NDs development in psoriatics, although it surely requires further research. GA might correspond with neuroinflammation in psoriasis. Systemic antipsoriatic therapy could be studied in order to improve cognitive impairment through lowering NDs biomarkers in some cases.

## 1. Introduction

Psoriasis is a common, chronic, inflammatory dermatosis which affects approximately 125 million people worldwide [1]. It is characterized by the increased proliferation of epidermal cells and immunological disturbances [2]. As psoriasis is associated with multiple comorbidities, scientists introduced a concept of psoriasis as a systemic disorder, affecting many more body organs than just skin [3]. It has been proved that patients with psoriasis suffer more often from metabolic syndrome, which is estimated to occur even in 65% of them [4]. Moreover, they are at increased risk of joint, intestinal, kidney and pulmonary diseases development, as well as psychiatric disorders [3]. The common background of psoriasis and its comorbidities is most of all metabolically induced inflammation, called ‘metaflammation’, but also genetic factors, similarly disturbed immunological and metabolic pathways or several bioactive substances released from the adipose tissue [5] Research shows that an important link in the pathogenesis of psoriasis might be oxidative stress, which occurs due to the imbalance between the reactive oxygen species and antioxidants, which in consequence leads to lipid peroxidation, DNA alterations and the secretion of proinflammatory cytokines [2,6].

Neurodegenerative diseases (NDs) are characterized by the progressive loss of nervous tissue [7]. There is an association between NDs and advanced age, therefore it is suspected that the frequency of such diseases is going to increase due to the aging of the population [8]. The most common diseases considered among NDs are: Alzheimer’s disease (AD), Parkinson’s disease (PD), Huntington’s disease (HD) or amyotrophic lateral sclerosis (ALS) [7]. Apart from genetic predisposition, one of possible pathogenic paths is oxidative stress, which contributes to the death of neurons and the loss of nervous tissue [9]. A promising method for diagnostics of NDs are different biomarkers, which are substances indicating particular diseases and sometimes predicting their onset and course. An example of such markers could be fatty acid binding protein 7 (FABP-7), neurofilament light chain (NFL) and glutamic acid (GA) [10,11,12].

Fatty acid binding proteins (FABPs) are cytosolic proteins which protect cells from the damaging influence of the accumulation of long chain fatty acids [13,14]. So far, there have been nine FABPs described, of which several have been investigated in psoriasis. The role of liver, heart–muscle and adipocyte FABP isoforms (FABP-1,3,4, respectively) in the relationship between psoriasis and cardiometabolic disorders has been established inter alia by our team [14,15]. FABP-7, called brain FABP, is present in the brain tissue and plays an important role in the development of the nervous system and is suspected to take part in the storage and transport of docosahexaenoic acid (DHA) and eicosapentaenoic acid (EPA) [10,16,17]. FABP-7 has also been implicated in the neurodegenerative process. Increased serum concentration of FABP-7 is observed in patients with different NDs, especially AD and PD [10,18]. It is interesting to note that B-FABP is similar to H-FABP in 67% and the latter isoform has also been suspected to be involved in neurodegeneration [10,19].

GA is an excitatory neurotransmitter in the nervous system. It is involved in the cortical and cognitive functions [20]. In the natural setting the highest concentration of glutamic acid is observed inside the cells, and it is lower outside them, in the cerebrospinal fluid (CSF) or the blood [20]. The elevation of glutamate in the extracellular space results in abnormal signaling in synapses and excitotoxicity, which eventually leads to the death of neurons [21]. An important issue is also the diffusion of GA in the extracellular space, which affects glial cells and contributes to neuroinflammation, a crucial phenomenon of neurodegeneration where neurons secrete increased amounts of proinflammatory cytokines [21]. Improper GA homeostasis has been suggested in the pathogenesis of AD, PD, HD or ALS [22]. Its increased concentration is observed in the substantia nigra of patients with PD, as well as their serum, especially at the beginning of the disease [12].

NFL is one of the neurofilaments subunits which are a compound of the neurons’ cytoskeleton, which takes part in their growth and intracellular transport [11]. When the axon damage occurs, NFL appears in the CSF and then in blood, therefore increased serum concentration of NFL may indicate advanced axons degeneration [11]. It is worthy of note that there is a proportional correlation between the degree of damage and the concentration of NFL [11]. NFL is released due to any axon injury, for instance also in cerebrovascular disorders or after trauma [23], but most importantly it is associated with different NDs, including AD, ALS or HD [11], among others. NFL has been assessed in psoriatic patients once and it turned out to be elevated [24].

Psoriasis and NDs share some common pathogenetic background, especially genetic and metabolic factors or oxidative stress. There are very few research investigating the possibility of NDs occurrence in psoriatic patients and they usually concern the actual incidence rate of NDs in such patients [23,25,26,27,28], not the search for predicting markers of such disorders. The mentioned phenomena indicate that psoriatics could actually be at an increased risk for the development of NDs. To the best of our knowledge, FABP-7 and GA have never been studied in psoriasis before and NFL has only been studied once [24]. Thus, we aimed to explore their potential role in the interplay between psoriasis and NDs along with relationships with clinical and laboratory parameters. Furthermore, we evaluated the impact of two systemic antipsoriatic drugs on the proteins levels and we aimed to elucidate whether they exert a protective effect on cognitive impairment in psoriatics.

## 2. Materials and Methods

This correlational study enrolled 60 patients (21 females and 39 males) with a flare of plaque-type psoriasis, at median age of 57 (19–80) years old and compared them with 30 sex-, age- and BMI-matched volunteers without skin diseases. All participants signed informed written consents before initiation. None of the patients or controls was under any dietary restriction or was taking medications for at least three months before the enrollment. The exclusion criteria comprised other types of psoriasis, chronic inflammatory diseases and cardiometabolic, autoimmune or oncological comorbidities. No patient was diagnosed with the ND at the time of study performance. Body mass index (BMI) was calculated as weight/height^2^ (kg/m^2^). The psoriasis area and severity index (PASI) was assessed by the same person in all patients. The investigated group was divided into three subgroups depending on skin lesion severity: mild (PASI I) < 10 points, moderate (PASI II) between 10 and 20, and severe (PASI II) > 20. All patients were further subdivided into groups according to BMI: BMI I was related to normal-weight (BMI 18.5–24.9) and included 12 persons; in the second group, BMI II, overweight (BMI 25–29.9) was noted in 13 psoriatic patients; and BMI II, obesity (BMI > 30) was observed in 10 patients. We also divided patients for short-lasting (<20 years) and long-lasting (>20 years) psoriasis duration. Laboratory tests including C-reactive protein (CRP), complete blood count (CBC), serum fasting glucose, total cholesterol (Chol), high-density lipoproteins (HDL), low-density lipoproteins (LDL), triacylglycerol (TGs), and transaminases (AST, ALT) were performed before treatment. The patients received two systemic treatment options: 15 subjects received methotrexate (MTX) 15 mg/week using folic acid supplementation (15 mg/week, 24 h after MTX intake) and 20 subjects received acitretin (0.5 mg/kg/day). The therapy lasted 12 weeks. The choice of particular drug was determined based on the recommendations of psoriasis management written by the Polish Dermatology Society [29]. The following factors have been taken into account: psoriasis severity (expressed by PASI), life quality (expressed by DLQI), body surface area (BSA), results of laboratory or imaging investigations, contraindications for particular drugs, and joints involvement.

The study was approved by the Bioethical Committee of the Medical University in Bialystok (number: R-I-002/429/2017) and was in accordance with the principle of the Helsinki Declaration.

### 2.1. Serum Collection

Fasting blood samples were collected from volunteers from the control group and patients before and after 12 weeks of treatment using vacutainer tubes with a clot activator. Samples were centrifuged at 2000× *g* for 10 min and preserved at −80 °C until analyses. FABP-7, NFL and GA levels were measured using an enzyme immunoassay kit supplied by Cloud Clone^®^ Houston, TX, USA (SEB277Hu, SEE038Hu, CES122Ge). Optical density was read at a wavelength of 450 nm. The concentrations were measured by interpolation from calibration curves prepared with standard samples supplied by the manufacturer. All the laboratory investigations were performed by the same person in standardized laboratory settings.

### 2.2. Statistical Analysis

Normality of distribution was tested using the Shapiro–Wilk W test. The normally distributed data were analyzed using a one-way analysis of variance (ANOVA) and shown as mean ± SD. The non-Gaussian data were presented as median (full range) and analyzed using the non-parametric Kruskal-Wallis test. The Student’s *t*-test or nonparametric Mann–Whitney test were used to compare differences between the psoriasis group and the control group. The correlations were analyzed using Spearman’s Rank correlation analysis. Statistical analysis was conducted using the GraphPad Prism 9.20 software (GraphPad Prism 9, 20 Software, La Jolla, San Diego, CA, USA). The differences were deemed statistically significant when *p* < 0.05.

## 3. Results

Baseline characteristics of patients and controls are presented in Table 1. There was no statistically significant difference between patients and controls in terms of age and BMI.

### 3.1. FABP-7

**Figure 1:**
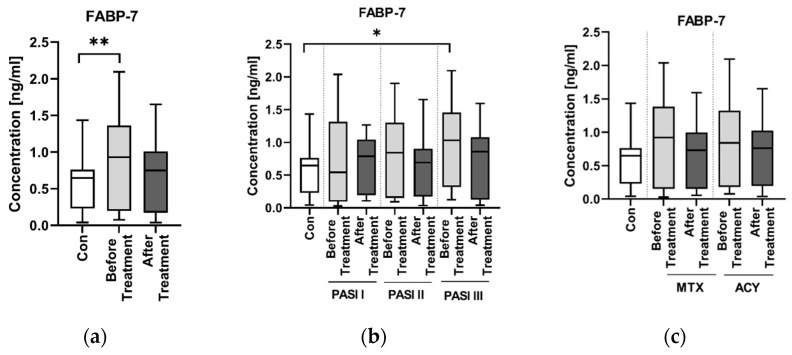
Concentration of FABP-7 in patients (before and after the treatments) and controls (**a**); Concentrations of FABP-7 in patients (before and after the treatment) according to PASI division, as well as in controls (**b**); FABP-7 concentrations in patients (before and after the treatment with methotrexate, MTX, or acitretin, ACY) and controls (**c**). ** means statistically significant difference with *p* < 0.01. * means statistically significant difference with *p* < 0.05.

The concentration of FABP-7 in patients before the treatment was higher than in the controls and the difference was statistically significant (*p* < 0.01) (Figure 1a).

There was no correlation between the FABP-7 concentration and age, BMI or PASI (NS) (Table 2).

FABP-7 concentrations were higher in subgroups with higher PASI, although the difference between the groups was not significant (NS) (Figure 1b). There was a significantly higher concentration of FABP-7 in the PASI III subgroup before the treatment, however, compared to the controls (*p* < 0.05) (Figure 1b).

**Figure 2:**
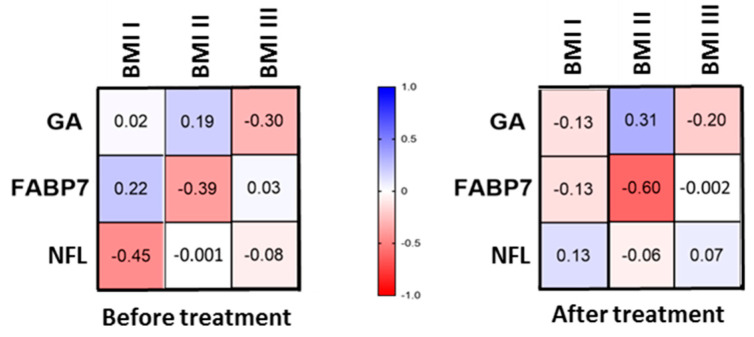
Division of patients before and after the treatment and into three subgroups according to BMI and correlations with FABP-7, GA and NFL concentrations.

After division into two groups before and after the treatment and three subgroups according to BMI, we found no correlations of FABP-7 with BMI (Figure 2).

There was no correlation between the FABP-7 concentration and laboratory parameters in general (NS) (Table 3).

We also looked for correlations between FABP-7 and laboratory parameters inside particular subgroups of patients divided according to PASI and according to BMI (Figure S1 in Supplementary Files) but there were no statistically significant observations (NS).

**Figure 3:**
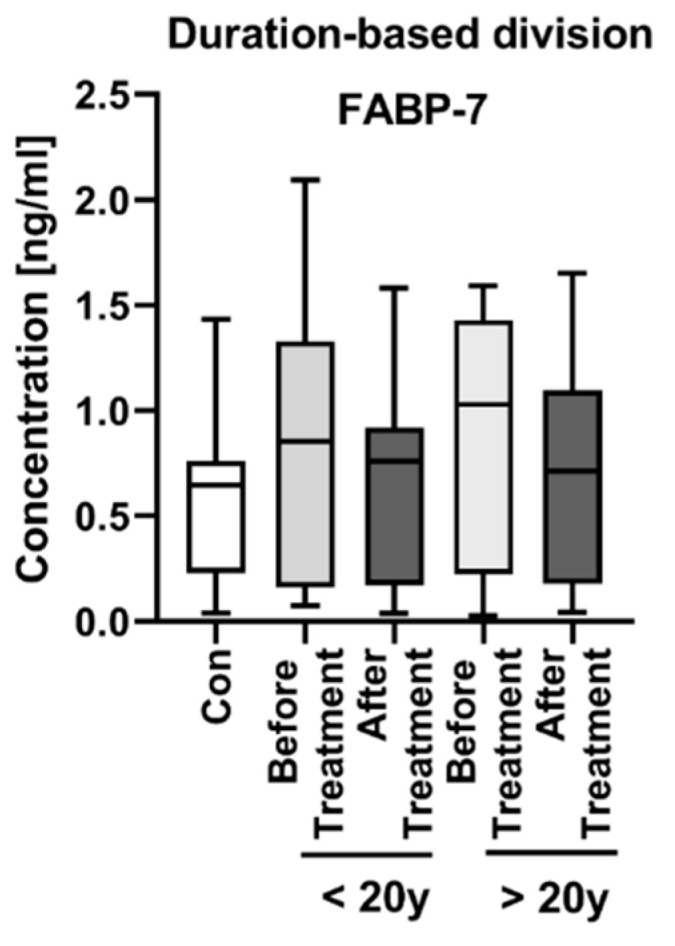
FABP-7 concentration in subgroups of patients after division according to the duration of the dermatosis–less or more than 20 years, with division before and after the treatment.

We also performed the division of psoriatics according to the duration of the dermatosis–less or more than 20 years–and looked for changes in FABP-7 concentrations before and after the treatment (Figure 3) and correlations with laboratory parameters (Figure S2 in Supplementary Files). The FABP-7 concentration was higher in patients with long-lasting psoriasis, although we did not observe any significant changes or correlations inside these subgroups (NS).

After the treatment, FABP-7 concentration decreased, although insignificantly (NS), and continued to stay higher than in the controls (Figure 1a). There was no difference in the concentration of FABP-7 between the groups divided according to the administered antipsoriatic drug (NS) (Figure 1c).

There was a negative correlation between FABP-7 and GA concentrations (R = −0.369, *p* < 0.006).

### 3.2. GA

**Figure 4:**
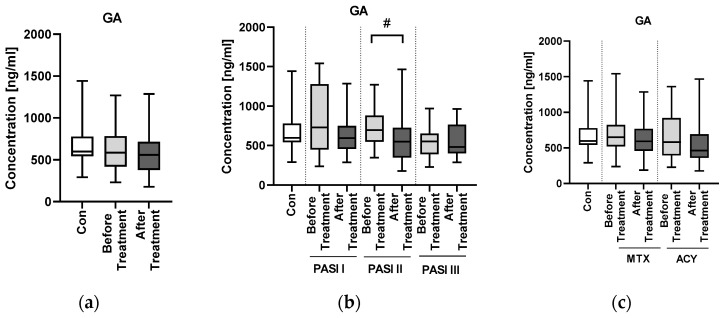
Concentration of GA in patients (before and after the treatments) and controls (**a**); Concentrations of GA in patients (before and after the treatment) according to PASI division, as well as in controls (**b**); GA concentrations in patients (before and after the treatment with methotrexate, MTX, or acitretin, ACY) and controls (**c**). # means statistically significant difference between patients before and after the treatment in PASI II subgroup with *p* < 0.05.

There was no significant difference in the concentration of GA between the patients and controls (NS) (Figure 4a).

There was no correlation between the GA concentration and age, BMI or PASI (NS) (Table 2).

There was no significant difference in the concentrations of GA between the subgroups divided according to PASI (NS) (Figure 4b).

After division into two groups before and after the treatment and three subgroups according to BMI, we found no correlations of GA with BMI (Figure 2).

There was no correlation between the GA concentration and laboratory parameters in general (NS), except for the negative correlation with CRP (R = −0.27, *p* < 0.05) (Table 3).

We also looked for correlations between GA and laboratory parameters inside particular subgroups of patients divided according to PASI and BMI (Figure S3 in Supplementary Files). In the PASI I subgroup we found negative correlations between GA concentration and BMI and PLT (R = −0.65, R = −0.64, respectively; *p* < 0.05), as well as a trend for AST (R = −0.63, *p* = 0.056). In the BMI I subgroup we found a negative correlation between GA and CRP concentration (R = −0.55, *p* < 0.05).

**Figure 5:**
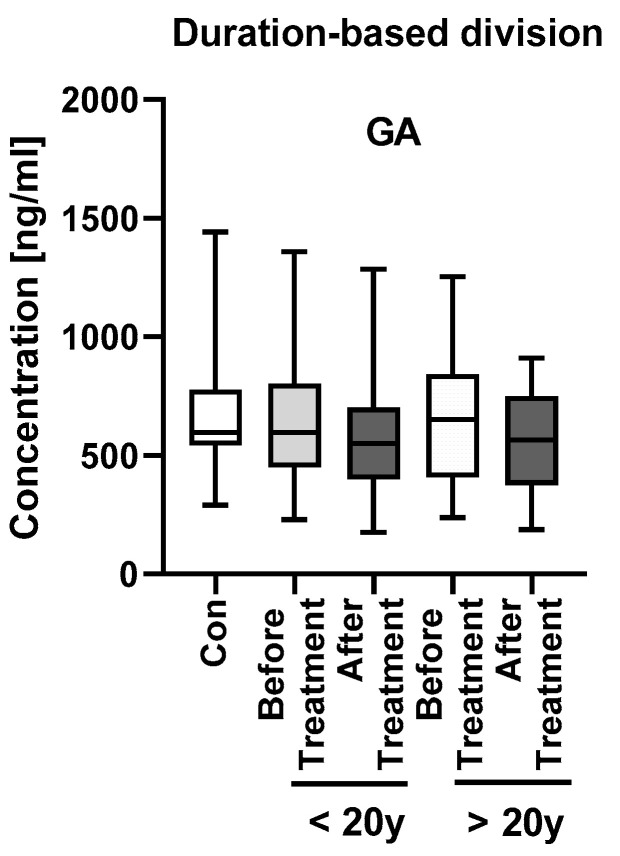
GA concentrations in subgroups of patients after division according to the duration of the dermatosis–less or more than 20 years, with division before and after the treatment.

As for the division of psoriatics according to the duration of the dermatosis (Figure 5, Figure S2 in Supplementary Files), patients with long-lasting psoriasis had higher GA concentrations, although insignificantly. We did not observe any significant changes or correlations inside these subgroups (NS), except for a negative correlation between GA concentration and PASI in patients with short-lasting psoriasis (R = −0.345, *p* < 0.05) and between GA and CRP concentrations in patients with long-lasting psoriasis (R = −0.542, *p* < 0.01).

After the administered treatment, GA concentration slightly, insignificantly decreased (NS) (Figure 4a). There was no significant difference in the concentration of GA between the subgroups divided according to the administered drug (NS) (Figure 4c).

### 3.3. NFL

**Figure 6:**
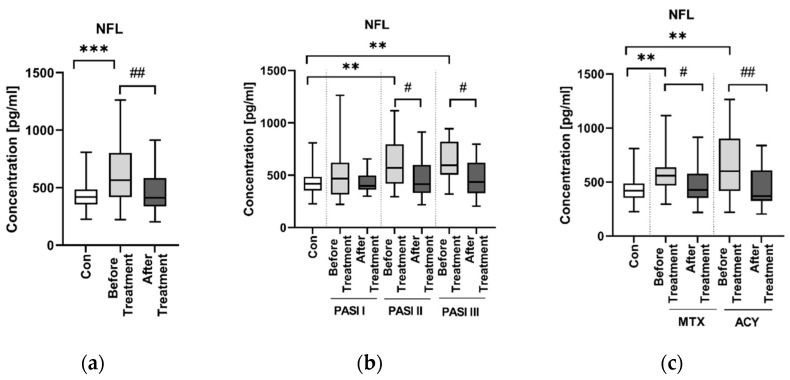
Concentration of NFL in patients (before and after the treatments) and controls (**a**); concentrations of NFL in patients (before and after the treatment) according to PASI division, as well as in controls (**b**); NFL concentrations in patients (before and after the treatment with methotrexate, MTX, or acitretin, ACY) and controls (**c**). ***/** means a statistically significant difference between the patients after the treatment and controls with *p* < 0.001/*p* < 0.01. ##/# means a statistically significant difference between the patients before and after the treatment with *p* < 0.01/*p* < 0.05.

There was a significantly higher concentration of NFL in patients than in controls (*p* < 0.001) (Figure 6a).

There was no correlation between the NFL concentration and age, BMI or PASI (NS) (Table 2).

The PASI division revealed that patients from the groups PASI II and PASI III had significantly higher concentrations of NFL before the treatment than the controls (both *p* < 0.01) (Figure 6b).

After division into two groups before and after the treatment and three subgroups according to BMI, we found no correlations of NFL with BMI (Figure 2).

There was no correlation between the NFL concentration and laboratory parameters in general (NS) (Table 3).

We also looked for correlations between NFL and laboratory parameters inside particular subgroups of patients divided according to PASI and according to BMI, and we noticed negative correlations between NFL concentrations and RBC and ALT in PASI III subjects (R = −0.635, *p* < 0.01; R = −0.473, *p* < 0.05, respectively), as well as between NFL and total cholesterol in the BMI III subgroup (R = −0.58, *p* < 0.01) (Figure S4 in Supplementary Files).

**Figure 7:**
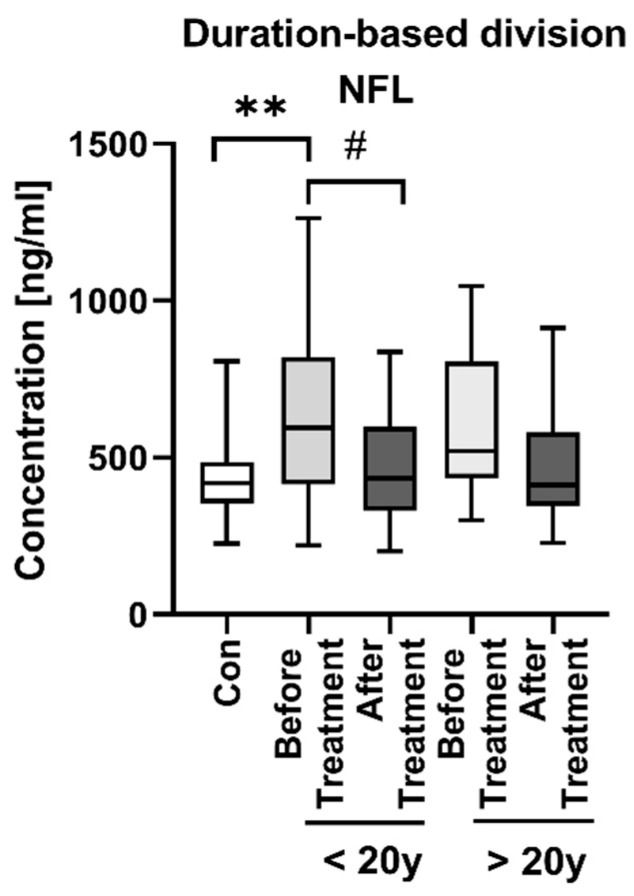
NFL concentrations in subgroups of patients after division according to the duration of the dermatosis (less or more than 20 years) with division before and after the treatment. ** means statistically significant difference between the patients before the treatment and controls with *p* < 0.01. # means statistically significant difference between the patients before and after the treatment with *p* < 0.05.

As for the division of psoriatics according to the duration of the dermatosis (Figure 7), we observed statistically significantly higher NFL concentration in patients before the treatment suffering from psoriasis < 20 years compared to controls (*p* < 0.01), but we did not observe any significant differences between the two subgroups in terms of short- and long-lasting psoriasis.

As for laboratory parameters after the division according to the duration of the disease, we found negative correlations between NFL concentrations and RBC in the group of short-lasting psoriasis (R = −0.357, *p* < 0.05) and triglycerides in the group of long-time psoriatics (R = −0.486, *p* = 0.018) (Figure S2 in Supplementary Files).

Regarding administered treatment, after the treatment NFL concentration decreased significantly compared to the status before the treatment (*p* < 0.01) (Figure 6a). In the subgroups PASI II and PASI III we observed a significant decrease in the NFL concentration after the treatment compared to the status before (both *p* < 0.05) (Figure S4 in Supplementary Files). There was a significant drop in the concentration of NFL after the introduction of both MTX and ACY, although it was more significant after ACY (*p* < 0.05; *p* < 0.01, respectively) (Figure 6c).

## 4. Discussion

Psoriasis was proved to be associated with different comorbidities [3], but the relationship with NDs has not been well established so far. As their frequency is expected to rise [8], it is all the more important to perform research in this field in order to find tools allowing for early detection. As for psoriasis, it is a very common dermatosis, which also decreases patients’ quality of life, and the current state of knowledge indicates that it could indeed be associated with NDs. There are very few original papers investigating this matter. They mainly focus on the actual incidence of NDs in subjects with psoriasis. In some of them an increased risk of AD in psoriatics [25,26] was found, while in others an increased risk of PD was noted [30,31]. On the other hand, in one study no shared genetic loci were found between psoriasis and ALS, AD or PD [27]. Nevertheless, more original studies are still needed. In the current paper we took a different approach, trying to assess the possible risk or susceptibility to NDs among psoriatics.

Apart from the role in the development in the nervous system, FABP-7 is also thought to play a protective role for neuronal tissue. Hara et al. suggested that it takes part in reactive gliosis and Senbokuya and Rui et al. believe that it influences astrocytes after brain and spinal cord injury [16,32,33]. It is possible that it protects DHA (which has antioxidant, anti-inflammatory and antiapoptotic properties) from the unfavorable peroxidation, which is a process whose products play an important role in the pathogenesis of NDs [34,35]. As we found FABP-7 to be significantly elevated in psoriatics compared to the healthy subjects, we conclude that it may suggest increased risk of NDs in this particular group of patients. FABP-7 has been reported to be increased in patients with AD or PD [10,17], so perhaps our finding is a clue that psoriatics are indeed at greater risk of such NDs. As nobody has ever performed a similar investigation, we have no data for comparison, but we believe it should be verified on a larger cohort and we are aware that our investigation should be treated as preliminary but still innovative.

FABP-7 concentrations were higher in subgroups with more intense psoriasis severity expressed by PASI, although the significant difference between the groups was observed only between PASI III and controls. As FABP-7 did not correlate directly with skin lesion severity in general, but was significantly increased in most diseased subjects, we might conclude that psoriasis severity does not influence the risk of NDs in psoriatic patients with the mild or moderate form, but patients with severe psoriasis might be at higher risk of NDs development and FABP-7 might serve as a potential indicator of it.

FABP-7 seemed to be independent of the age and BMI of patients. We also did not find any correlations between FABP-7 concentrations and laboratory parameters, even after division according to PASI or BMI. Moreover, it appears that the time of suffering from psoriasis does not influence FABP-7 concentrations in patients, so the potential risk of NDs development is independent of the psoriasis duration and other factors that affect these relationships.

After the total treatment, serum FABP-7 concentration decreased, although insignificantly, and stayed higher than in the controls. None of the administered classic antipsoriatic agents showed any advantage than the other on the influence of FABP-7 concentration, therefore the choice of the classic systemic therapy in psoriatics with elevated FABP-7 concentration seems voluntary. We did not manage to find any information about the influence of methotrexate or acitretin on FABP-7 serum concentrations in the available literature.

As for GA, research has shown that increased release and/or decreased uptake of glutamate leads to abnormal neuronal calcium levels, followed by oxidative stress, mitochondrial dysfunction, protein turn-over dysregulations and neuroinflammation [8]. As these mechanisms were proved to be involved in the pathogenesis of NDs, they were deeply investigated and evaluated in relation to possible therapeutic options [8]. Elevated levels of serum GA have been demonstrated in different disorders [20]. In our study there was no significant difference in GA concentration between the patients and controls. There was also no correlation between GA concentration and age, BMI or PASI. The literature suggests increased serum concentrations of GA in patients with NDs, especially PD [27], but unfortunately we were not able to confirm such observations. GA was significantly negatively correlated with CRP level in our psoriatic patients, and more in those with long-lasting disease or normal weight. An excessive amount of GA contributes to the neuroinflammation during which several cytokines are released and lead to GA receptors’ upregulation and its increased uptake, which further increases its toxicity [21]. Hypothetically, our observation could therefore correspond with the following decrease of GA after its uptake and constantly sustained inflammatory condition. Nevertheless, perhaps in psoriatic patients this relationship could have a different explanation, and GA could act alternatively in such patients, even presenting another role; for instance, as we noted a negative correlation between GA and CRP, the simplest explanation could be that it serves as anti-inflammatory agent in psoriatics. It is just a suspicion, however, that would have to be investigated on larger cohorts.

After the treatment, GA concentration slightly, insignificantly decreased, and there was no difference between particular systemic antipsoriatic agents in the influence on GA concentrations. We found no reports regarding the potential influence of acitretin on GA concentrations, but we managed to find some information on methotrexate. As a matter of fact, methotrexate itself may cause neurotoxicity due to intrathecal or systemic administration, independently of the dose (high/low) [36]. One of the potential paths involved in this side effect is that glutamate may be released from methotrexate cleavage and then lead to neurotoxic complications [37]. Whether there is a direct relationship between CSF and blood GA concentration is uncertain, because some of researchers support this theory, whereas others deny it [20]. Further research is definitely needed.

NFL has been proved to be a marker of NDs but, as it is released due to any axon injury [11], it seems to be rather more sensitive than the specific marker, therefore it appears to be useful in distinguishing NDs from non-NDs or between neurological diseases that vary in the extent of large myelinated axon damage [11]. In our study, NFL concentration was significantly higher in patients in comparison to the controls. This observation is important, since research has shown that subjects with NDs, especially AD, tend to have higher concentrations of NFL than persons without cognitive impairment. NFL concentration was found to also be associated with cognitive, biochemical and imaging hallmarks of this disease. This is why the authors suggested NFL as a potential noninvasive marker for AD development [38]. NFL is not specific for AD, however. Its higher concentrations were also observed in other NDs, such as PD, multiple sclerosis (MS), HD, ALS, frontotemporal dementia or Creutzfeldt-Jakob disease [39,40]. Moreover, NFL concentration seems to increase a few years before the onset of NDs [40], which might allow for the identification of subjects at risk and prevent disease progression and irreversible complications. NFL serum concentration has also been associated with the severity of symptoms of different NDs, e.g., it positively correlates with the Unified Parkinson’s Disease Rating Scale (UPDRS) in PD, motor and cognitive impairment in HD, Alzheimer’s Disease Assessment Scale-cognitive subscale (ADAS-cog) in AD and negatively with Mini-Mental State Examination (MMSE) in AD [11]. NFL is even described as one of the most promising markers in clinical practice and research in the near future [11]. An interesting issue is the genetic link between psoriasis and NDs. It has been proved that subjects with apolipoprotein E polymorphism have an increased risk of development of AD in the first place, but also PD or HD [41]. At the same time, the same polymorphism has been demonstrated in the pathogenesis of psoriasis [41,42]. NFL was also described as a potential marker for monitoring persons with genetic risk factors of AD. It appears that there is a correlation between the serum NFL and estimated time to the onset of symptoms in autosomal dominant AD mutation carriers [11]. Taken altogether, perhaps NFL could therefore become another marker of greater risk for NDs in psoriatics. We managed to find one study which aimed to assess NFL concentrations in psoriatic patients, but it was performed on a smaller group than ours [24]. Similar to our results, serum NFL concentrations were increased in patients compared to the control group [24], which supports our outcomes.

Despite the fact that there was not a direct correlation between NFL concentration and PASI in the whole group of patients, psoriatics with moderate and severe psoriasis tended to have significantly higher NFL concentration than subjects without the dermatosis. In the mentioned paper by Okan et al., contrary to our study, NFL was positively correlated with severity of psoriasis in PASI [24]. As NFL has never been studied only once before in psoriasis [24], we have little data for comparison, but it seems that psoriasis severity may be a factor affecting the neurodegenerative process, since it leads to elevation of NFL. Presumably, psoriatics with a more severe form require special attention or prevention regarding the possible development of NDs.

We found no correlations of NFL with BMI, so it appears that the body weight does not play an important role in the risk of cognitive complications in psoriatics. There is evidence that BMI, and hence blood volume, may affect NFL concentrations, e.g., in MS [43], but in our study, there was no significant difference between NFL concentrations in particular BMI groups, and therefore we ruled out this possible factor affecting our outcomes. The other study which assessed NFL in psoriatics revealed a positive correlation between NFL concentration and BMI [24]. As for a potential association with the age of subjects, the literature mentions that NFL concentration is probably dependent on age: the older the age, the higher the release of NFL from the axons [11]. In our study we did not find correlation of NFL serum concentration with age.

In the whole group of patients in general we did not notice any significant correlations between NFL concentration and laboratory parameters. Nevertheless, we would like to pay attention to the negative correlation between NFL and RBC in the patients with severe psoriasis. Erythrocytes take part in oxygen transport and tissue oxygenation balance. The RBC level may decrease due to different pathological conditions because of their sensitivity to reactive oxygen species [44]. Oxidative stress is an important link in the pathogenesis of both psoriasis and NDs, therefore this result remains consistent. Although we did not observe associations between lipid parameters and NFL, Okan et al. found they were positively correlated [24].

As for the influence of the duration of psoriasis on the NFL concentration, it seems that there is no relationship. Indeed, subjects with psoriasis had significantly higher NFL concentrations, but after the division, only those who suffered from this dermatosis less than 20 years. Therefore, the duration of psoriasis apparently does not directly affect the risk of neurodegeneration and is not a crucial factor in this interplay.

The therapy caused a significant decrease in NFL concentration compared to the values before the treatment. Moreover, both administered systemic agents induced such a significant drop, particularly acitretin. Therefore, we may assume that in patients with higher NFL concentrations, acitretin should be the drug of choice and may be beneficial not only in the treatment of skin lesions but also in the improvement of cognitive impairment, which seems to be consistent with the literature. NFL has been already used as a marker of therapeutic efficacy [45].

There are several reports on the beneficial influence of antipsoriatic agents on the mental and physical condition in patients with AD and PD. However, they usually concern biologics. For instance, there was evidence of the improvement observed in AD patients after administration of anti-TNFα agents for psoriasis [26]. Several other studies indicated the beneficial influence of dimethyl fumarate in AD, PD and ALS, which is also used in psoriasis treatment [46]. Unfortunately, we enrolled only patients on methotrexate or acitretin into our study, and there were no patients treated with an anti-TNFα agent or dimethyl fumarate in this group, so we cannot compare our results. We did not find a single piece of information about methotrexate affecting NDs course or development risk. We did find information on retinoids, however. Acitretin, which is widely used in psoriasis therapy because of its influence on epidermal cells proliferation and differentiation, seems to be potentially beneficial in AD, since it inhibits amyloid synthesis and leads to cognitive improvement in AD mice models [47]. In our study, a significant drop in the investigated protein concentrations was observed only for NFL. It was observed after the treatment in the whole group of patients, as well as in patients who received only systemic therapy, both with methotrexate and acitretin.

We would like to point out some of the limitations of the study. First, our study took a single-centered approach. The participants were only of Caucasian ethnicity. We measured the protein concentrations twice (before and after the treatment) and perhaps more measurements over the time would be more valuable. On one hand, our study is the first of its kind, which brings novelty to the current state of knowledge on psoriasis, while on the other hand it is a limitation, since we have no similar research to compare it with. Our results should be treated as preliminary, but they still point to future directions for studies. We plan to extend our research in cooperation with neurologists and psychiatrists in order to optimize the diagnostic approach.

## 5. Conclusions

We investigated three NDs serum markers in order to find out whether they could indicate greater risk of NDs development in patients with psoriasis. Of note, this method of testing seems relatively noninvasive and cheaper than other complicated tests, including imaging or genetic investigations, and there is a real chance to apply such methods in a daily practice. As we found two of three investigated markers significantly elevated in psoriatics, namely FABP-7 and NFL, which are present in the brain, we conclude that they may be indicators of increased risk of NDs in these patients, although it surely requires further research. Moreover, as NFL has turned out to be an ND onset and severity marker, it could perhaps be similarly applied in psoriatic patients’ comorbidity monitoring. Psoriasis severity seems to not affect the risk of NDs, however cognitive impairment might be more prevalent in severe psoriatics and FABP-7 and NFL could be a biomarker for them. GA may correspond with neuroinflammation in psoriasis but it surely requires further in-depth research. The duration of psoriasis seems to not affect the risk of NDs, but since two biomarkers were higher, although insignificantly, in patients with long-lasting psoriasis, it should be perhaps investigated on larger cohorts. Systemic antipsoriatic treatment turned out to be beneficial in lowering ND biomarker concentrations in some cases, so we may suspect that antipsoriatic therapy could improve cognitive impairment or prevent it, due to the pleiotropic activity of some agents, which is consistent with the literature. Our results seem interesting and surely innovative, nevertheless further research is required to establish definite associations between psoriasis and NDs.

## Figures and Tables

**Table 1:** Baseline characteristics of patients and controls.

Parameter	Controls (*n* = 30)	Patients (*n* = 60)
Sex (M/F)	10/20	39/21
Age [years]	52.5 (25–64)	57 (19–80) NS
Height [cm]	166 (156–186)	175 (154–190) NS
Weight [kg]	67.5 (50–133)	83 (54–136) NS
BMI	24.6 (20–41)	27.1 (17–44.4) NS

NS, non-significant, M/F, male/female, BMI, body mass index.

**Table 2:** Correlations between PASI, BMI and FABP-7, NFL and GA concentrations.

Protein	PASIR (*p* Value)	BMIR (*p* Value)
GA	0.188 (0.158)	−0.069 (0.604)
FABP-7	−0.476 (1604 × 10^−4^)	0.149 (0.265)
NFL	−0.091 (0.499)	−0.051 (0.593)

**Table 3:** Correlations between FABP-7, GA, NFL and laboratory parameters.

	AST R (*p*)	ALT R (*p*)	GLU R (*p*)	CRP R (*p*)	Chol R (*p*)	TG R (*p*)	RBC R (*p*)	WBC R (*p*)	PLT R (*p*)
GA	−0.05 0.711	−0.14 0.318	0.03 0.830	−0.27 0.049 *	0.24 0.086	0.09 0.498	0 0.989	−0.14 0.302	−0.14 0.363
FABP-7	0.03 0.837	0.08 0.579	0.05 0.701	−0.02 0.873	−0.01 0.949	−0.01 0.964	−0.24 0.086	0.05 0.737	0.08 0.981
NFL	−0.21 0.109	−0.02 0.891	−0.02 0.904	−0.03 0.832	0.1 0.469	0.19 0.163	−0.12 0.374	−0.13 0.327	−0.12 0.782

* bold font means a statistical significance of *p* < 0.05. TGs, triglycerides; CRP, C-reactive protein; ALT, alanine transaminase; AST, asparagine transaminase; GLU, fasting glucose; Chol, total cholesterol; RBC, red blood cells; WBC, white blood cells; PLT, platelets.

## Data Availability

Data available on the request.

## Supplementary Materials

The following supporting information can be downloaded at: https://www.mdpi.com/article/10.3390/jcm11092430/s1. Figure S1. Division into three groups according to PASI and BMI and correlations between FABP-7 and laboratory parameters inside each group. Figure S2. Correlations of FABP-7, NFL and GA concentration with laboratory parameters in subgroups of patients after division according to the duration of the dermatosis–less or more than 20 years. Figure S3. Division into three groups according to PASI and BMI and correlations between GA and laboratory parameters inside each group. Figure S4. Division into three groups according to PASI and BMI and correlations between NFL and laboratory parameters inside each group.
