# Supplementary material for: Fatty Acid-Binding Protein 7 (FABP-7), Glutamic Acid and Neurofilament Light Chain (NFL) as Potential Markers of Neurodegenerative Disorders in Psoriatic Patients—A Pilot Study
Source: J Clin Med. 2022 Apr 26;11(9):2430. doi: 10.3390/jcm11092430 (PMC9105148; doi:10.3390/jcm11092430)
Supplement: Supplementary file 1 [file jcm-11-02430-s001.zip › jcm-1650746-supplementary.pdf]

Supplementary files

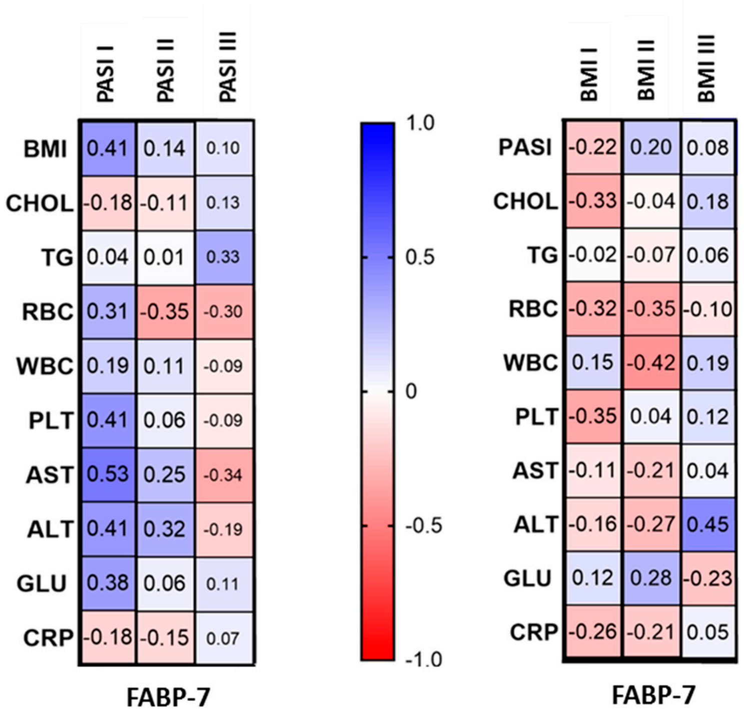

Figure S1. Division into three groups according to PASI and BMI and correlations between FABP-7 and laboratory parameters inside each group.

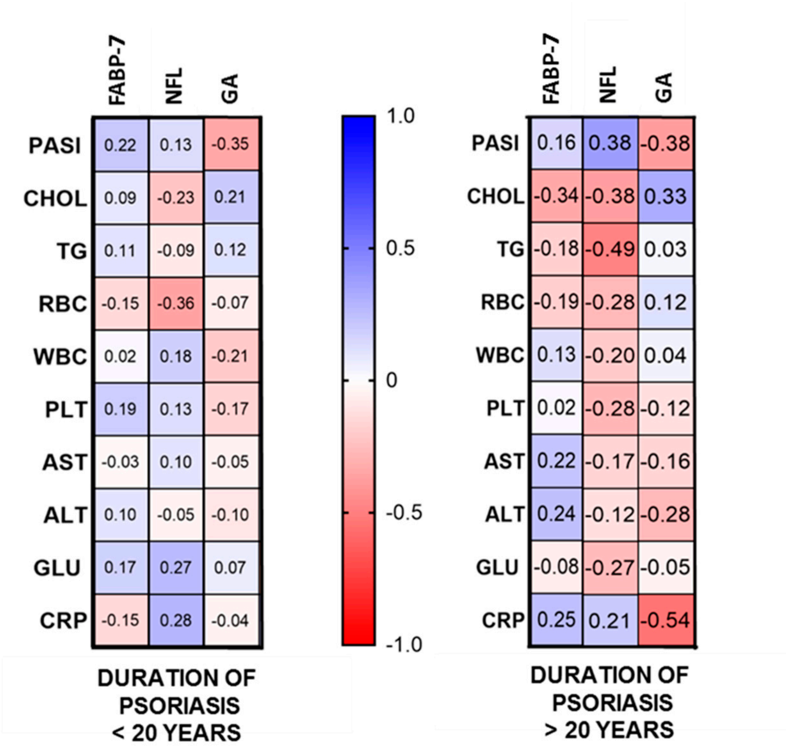

Figure S2. Correlations of FABP-7, NFL and GA concentration with laboratory parameters in subgroups of patients after division according to the duration of the dermatosis – less or more than 20 years.

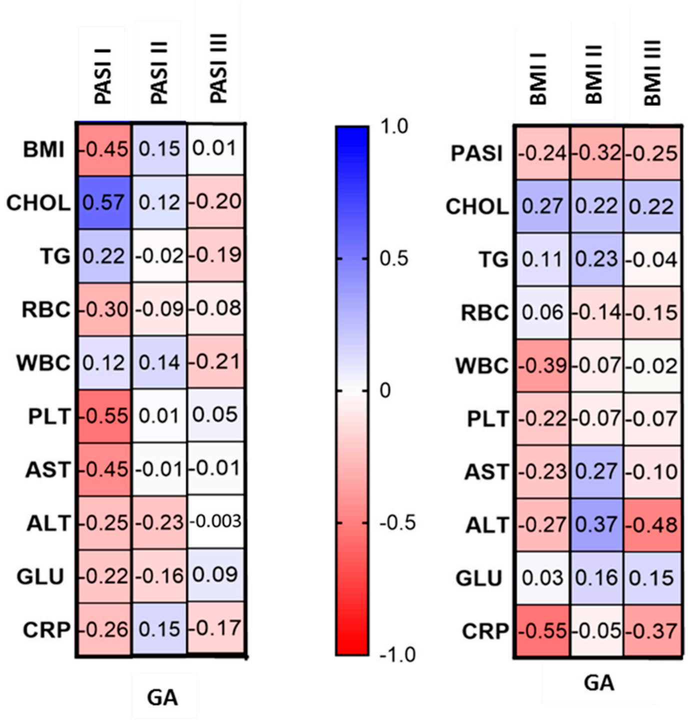

Figure S3. Division into three groups according to PASI and BMI and correlations between GA and laboratory parameters inside each group.

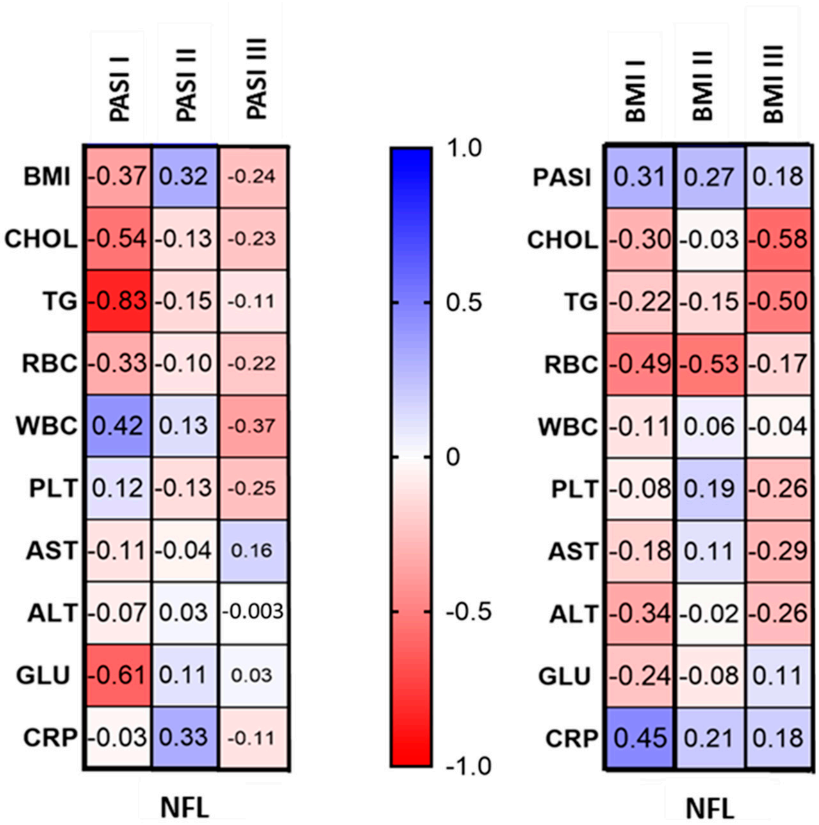

Figure S4. Division into three groups according to PASI and BMI and correlations between NFL and laboratory parameters inside each group.
